# Heart Diseases Recognition Model Based on HRV Feature Extraction over 12-Lead ECG Signals

**DOI:** 10.3390/s24165296

**Published:** 2024-08-15

**Authors:** Ling Wang, Tianshuo Bi, Jiayu Hao, Tie Hua Zhou

**Affiliations:** Department of Computer Science and Technology, School of Computer Science, Northeast Electric Power University, Jilin 132013, China; smile2867ling@neepu.edu.cn (L.W.); 2202200985@neepu.edu.cn (T.B.); 2202100964@neepu.edu.cn (J.H.)

**Keywords:** heart rate variability, feature extraction, spectral magnitude quantization, ECG signal processing, random forest, heart disease recognition

## Abstract

Heart Rate Variability (HRV) refers to the capability of the heart rhythm to vary at different times, typically reflecting the regulation of the heart by the autonomic nervous system. In recent years, with advancements in Electrocardiogram (ECG) signal processing technology, HRV features reflect various aspects of cardiac activity, such as variability in heart rate, cardiac health status, and responses. We extracted key features of HRV and used them to develop and evaluate an automatic recognition model for cardiac diseases. Consequently, we proposed the HRV Heart Disease Recognition (HHDR) method, employing the Spectral Magnitude Quantification (SMQ) technique for feature extraction. Firstly, the HRV signals are extracted through electrocardiogram signal processing. Then, by analyzing parts of the HRV signal within various frequency ranges, the SMQ method extracts rich features of partial information. Finally, the Random Forest (RF) classification computational method is employed to classify the extracted information, achieving efficient and accurate cardiac disease recognition. Experimental results indicate that this method surpasses current technologies in recognizing cardiac diseases, with an average accuracy rate of 95.1% for normal/diseased classification, and an average accuracy of 84.8% in classifying five different disease categories. Thus, the proposed HHDR method effectively utilizes the local information of HRV signals for efficient and accurate cardiac disease recognition, providing strong support for cardiac disease research in the medical field.

## 1. Introduction

Cardiovascular Diseases (CVDs) are among the major threats to human health. Annually, they rank as the primary cause of death, making them one of the deadliest diseases globally. Most deaths from cardiovascular diseases are sudden; thus, patients do not have the opportunity to receive timely medical assistance [1]. Heart disease can stem from a variety of factors, such as hypertension, obesity, elevated cholesterol levels, smoking, and poor dietary choices. Daily combined modern biosensor monitoring for heart diseases will aid in initial diagnosis and prevention and in the identification of appropriate treatment [2]. Heart disease patients’ health management based on early identification and daily monitoring is critical [3].

The ECG records the heart electrical signals, collecting valuable information to aid in understanding the activity of the cardiovascular system. As a non-invasive diagnostic tool, it is widely employed for monitoring and diagnosing heart conditions. It can identify early signs of heart disease and provide appropriate treatment. The ECG provides cardiologists with all the necessary information about the heart’s condition, making it an effective tool for identifying various heart diseases [4]. Recent advances in machine learning have shown that ECG signals are useful for detecting heart disease. At present, the most common methods of processing the characteristics of ECG signals involve detection of the position of T waves, P waves, and QRS waves [5]. However, ECG signal noise effects could interfere with T, S, R, P, and Q wave detection accuracy [6]. At present, most methods for processing ECG signal characteristics involve detecting the positions of the T wave, P wave, and QRS complex. However, the QRS complex, particularly the R wave, is fundamental for detecting HRV signals, making it crucial to accurately identify the R peaks.

Furthermore, continuous advancements in medical technology have significantly contributed to the development of machine-learning systems. These technological improvements have enabled more accurate and efficient analysis of complex medical data, leading to earlier diagnosis and more personalized treatment options [7,8]. Under these studies, this paper uses the relevant features extracted in different heart diseases and uses machine-learning algorithms to classify these extracted features, with the common goal of improving diagnostic accuracy. Among these features, one of the most prominent is the Heart Rate Variability (HRV) signal. HRV sequences, which are consecutive heartbeat intervals over a period of time, provide valuable insights into cardiovascular health. Heart Rate Variability (HRV) can effectively predict the health status of an individual, as the heart rhythm is regulated by various physiological processes [9] and is considered a good indicator of various heart abnormalities.

The Autonomic Nervous System (ANS) plays a crucial role in regulating heart rate and other vital functions, making its analysis essential in understanding cardiovascular health. HRV analysis, which reflects the balance between the sympathetic and parasympathetic branches of the ANS, is particularly valuable for assessing heart conditions. Autonomic Nervous System (ANS) branch balance can be reflected by heart rate variability spectral analysis, and it can also be used as a monitoring tool for cardiovascular diseases in non-invasive techniques [10]. Guidelines have been established for the evaluation, physiological interpretation, and clinical application of resting heart rate variability, including three distinct spectral element descriptions: the High-Frequency 1 (HF1) spectrum, covering 0.15 to 0.25 Hz, the Low-Frequency (LF) spectrum, covering 0 to 0.15 Hz, and the Higher-Frequency 2 (HF2) spectrum, covering 0.25 to 0.4 Hz. The high-frequency component primarily reflects parasympathetic nervous activity. The low-frequency HRV is more complex, reflecting both sympathetic and parasympathetic activities, but is more indicative of sympathetic activity. HRV can indicate variations over the Autonomic Nervous System, thereby indicating the characteristics of various diseases.

Therefore, this paper aims to investigate the correlation between heart diseases and HRV patterns in electrocardiogram signals by extracting the pertinent features of HRV signals, exploring the differences in HRV characteristics among various heart diseases, and thus identifying and classifying different types of heart disease.

The following is the main structure of the paper: Section 1 describes the specific research background of the current heart disease identification monitoring technology; Section 2 identifies related work. Section 3 outlines the materials and methods employed in the study and the motivation behind the research; Section 4 specifies the HHDR model algorithm. Section 5 showcases the experimental analysis and interpretation of the findings. Section 6 offers an in-depth discussion; Section 7 summarizes the findings of the study.

## 2. Related Work

Heart disease detection via Electrocardiogram (ECG) signals has primarily focused on utilizing raw ECG data and various classification algorithms. In recent years, machine-learning techniques have shown significant potential in this area, with several studies exploring different approaches to enhance accuracy and reliability. One notable study by Shah and his team developed a model for predicting cardiovascular diseases using model-based supervised learning techniques, including Random Forest (RF), K-Nearest Neighbors (KNN), Decision Trees (DT), and Naive Bayes (NB), based on human bio-signals from ECG data. Their findings revealed that, among the models tested, the K-Nearest Neighbors algorithm achieved the highest precision, demonstrating its effectiveness in heart disease classification [11]. Similarly, Hasan and Bao’s team sought to refine the feature identification process for cardiovascular disease prediction. Their research indicated that the XGBoost classifier, when utilized with an ensemble wrapper method, could predict cardiovascular diseases with high accuracy. This study further underscores the growing importance of advanced machine-learning techniques in this field [12].

Building on the application of deep learning, Oh’s team utilized a U-net network to simultaneously detect R waves and arrhythmias. They employed an ECG signal autoencoder to diagnose conditions such as Right Bundle Branch Block (RBBB), Ventricular Premature Beats (VPB), Atrial Premature Beats (APB), Normal Sinus Rhythm (NSR), and Left Bundle Branch Block (LBBB) on the MIT dataset, achieving a classification accuracy of 97.32%, with R-peak classification accuracy reaching 99.3% [13]. For assisting arrhythmia diagnosis, Hou et al. proposed an LSTM-based autoencoder to select ECG features, achieving a specificity of 90.75%, sensitivity of 62.99%, and an average accuracy of 85.20% using a record-based cross-validation method [14]. This highlights the potential of deep learning in improving diagnostic precision for arrhythmias. Further exploring classification techniques, Smigiel and his team investigated disease classification across various categories, finding that convolutional networks with entropy features achieved the highest classification accuracy, underscoring the importance of feature engineering in model performance. Their work on Few-Shot Learning (FSL) with deep convolutional neural networks demonstrated the potential for these models to classify heart disease with high accuracy, even with limited data [15,16]. In related studies, Sieciński [17] conducted experiments comparing routine features of ECG, Seismocardiogram (SCG), and Gyroscopic Cardiogram (GCG) between patients with valvular heart disease and healthy individuals, providing further insights into diagnostic methodologies. Additionally, Ban et al. [18] designed and studied the use of linear and nonlinear HRV parameters to evaluate the Autonomic Nervous System (ANS). They extracted 36 features from ECG signals and implemented feature reduction and selection methods, ultimately achieving a model accuracy of 99%, demonstrating the effectiveness of these features in enhancing diagnostic accuracy. Together, these studies highlight the continued progress in utilizing sophisticated signal processing and techniques to improve the accuracy and efficiency of cardiovascular diagnosis.

Heart Rate Variability (HRV), regulated by the Autonomic Nervous System (ANS), is another critical area of research in heart disease detection. Li’s research team investigated how HRV can be monitored by smart devices, and what the public health implications are of this practice of monitoring HRV [19]. They found that HRV monitoring provides crucial insights into cardiac function and further reflects the health of the ANS. Additionally, using smart devices for long-term HRV monitoring with personal information can aid healthcare researchers in achieving individualized and precise monitoring. The study fully demonstrated the feasibility of HRV as a reference for cardiac activity. To fully verify the importance of HRV for identification purposes, Bahameish’s team integrated HRV signals with machine learning to classify mental health states, such as relaxation and stress. Their results indicated a high efficiency in distinguishing these states, with an F1 score of 86.3% for stress versus relaxation, and 65.8% for distinguishing a neutral state [20]. Razak’s [21] team explored methods for acquiring drivers’ HRV and developed machine-learning techniques to classify drivers’ cardiac health based on HRV data. The study found that the highest accuracy was achieved by decision-tree classifiers, reaching 92.86%, with K-nearest neighbors also performing well. Further research by Padovano et al. used ECG signals to study the relationship between heart disease and obstructive sleep apnea. The results illustrated the complex interplay between heart conditions and sleep disorders [22]. Mu et al. [23] proposed a deep-learning method for fatigue detection based on HRV feature extraction, which considered the sampling-efficient multi-head attention mechanism with contextual information calculation. In our previous study, we were also interested in fatigue status detection issues and proposed a multi-sensor fusion computing method based on EEG and ECG signals [24]. Moreover, the Emotion Quantitative Analysis (EQA) method is proposed to calculate semantic emotions onto valence-arousal two-dimensional domains, which is further used to mine the potential relationships between emotions and HRV signal responses [25]. In our previous study on the link between emotions and HRV responses, we proposed the Emotion Quantitative Analysis (EQA) method to objectively map emotions onto a valence-arousal, two-dimensional domain [25]. At the same time, we used physiological signals for the quantitative labeling of ECG signals, and accurately identified the 17-dimensional classification of ECG signals and emotions [26].

In conclusion, while previous studies have made significant progress in the use of HRV and ECG signals for heart disease detection, there remains a need for further research to harness the full potential of HRV signals and improve heart disease identification across a broader spectrum of cardiovascular conditions. Further research is needed to utilize HRV signals more effectively and to improve heart disease identification based on ECG signals.

## 3. Materials and Methods

### 3.1. Motivation

Heart Rate Variability(HRV) is a crucial metric for assessing fluctuations in heart rhythm, reflecting the autonomic nervous system’s regulation of cardiac function. Recent advancements in Electrocardiogram (ECG) signal processing technology have significantly improved our ability to analyze HRV features, providing valuable insights into various aspects of cardiac activity, such as heart rate variability, cardiac health status, and physiological responses. Despite these advancements, the accurate recognition of cardiac diseases remains a significant challenge.

This paper proposes a HRV-based method for heart activity recognition, focusing on the relationship between HRV fluctuations and cardiac activity changes, as well as relevant signal features. The method aims to deeply explore the uniqueness of HRV signal fluctuations among different heart diseases. The potential variability features are studied by rule extraction and other algorithms, and a Spectral Magnitude Quantification (SMQ) technique for extracting features is proposed to identify and evaluate the frequency band characteristics corresponding to various heart disease HRV signals. Finally, after extracting the features, the Random Forest (RF) classifier is deployed, and good classification results and high accuracy are obtained.

### 3.2. Dataset

PTB-XL is the dataset used in the research process and comprises 12-lead ECG waveforms collected from clinical environments, containing 21,836 records from 18,885 patients. This dataset has been reviewed by two cardiologists and is a multi-label dataset covering a wide range of diagnostic categories. For more details, see [27]. The distribution of data across various categories of heart diseases is shown in Table 1.

### 3.3. Heart Disease Identification Flowchart

**Figure 1:**
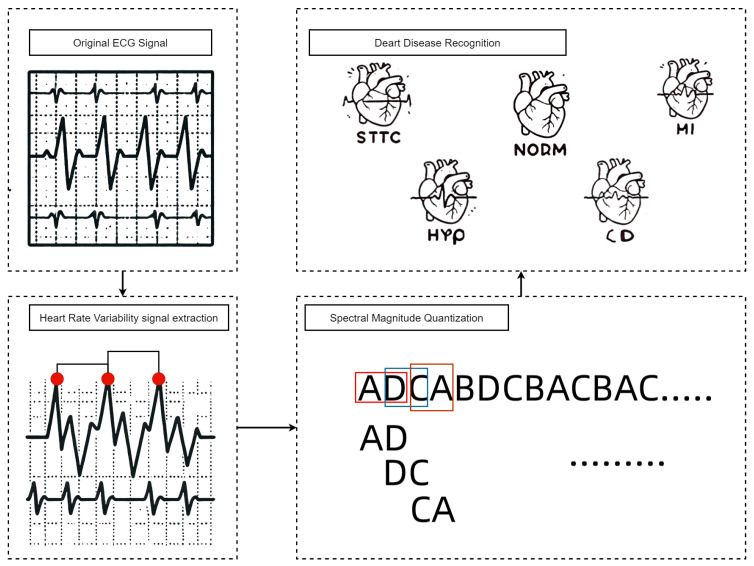
Heart disease identification flowchart.

ECG signals can more intuitively reflect abnormal cardiac activity, and the HRV values extracted from ECG signals can effectively indicate these abnormalities. Therefore, this paper focuses on collecting ECG signals using portable devices, extracting HRV signals for heart disease identification, and achieving accurate classification of abnormal heart rates to identify the corresponding heart diseases, as illustrated in Figure 1. From the raw ECG signals to HRV signal extraction (red points are R-peak points), the extracted HRV signals are quantified into a spectral magnitude sequence. The figure shows a spectral magnitude sequence (such as AD, DC, CA) set extracted using a sliding window of length 2. Finally, heart diseases are identified based on the matched rules. The identified heart categories are abbreviated as shown in Table 1 above.

## 4. HRV Heart Disease Recognition Model (HHDR)

Heart Rate Variability is a representation of the heartbeat interval, as well as the ECG RR interval. By analyzing HRV, crucial information about heart health can be obtained. Early identification of heart diseases is vital for prevention and treatment, and HRV analysis provides a non-invasive and convenient detection method.

Our proposed HHDR model effectively identifies changes in heart activity based on ECG signals, objectively detecting these variations. According to the PTB-XL dataset classification, we process the five categorized data types (NORM, STTC, HYP, CD, MI). Given that the dataset comprises 12-lead data, we treat each lead as a separate window, extracting the latent relationships within each window. Subsequently, we construct individual rule sets for each of the five data categories and correlate them with the original data.

By matching rules from the rule library, we extract features for each signal category. These extracted features are then analyzed using a random forest classifier to calculate the feature importance for each label category. We combine the top three features influencing each category to generate five new features, which are then used alongside the original features for classification using the random forest algorithm.The following sections introduce each part of the HRV Heart Disease Recognition Model (HHDR).

### 4.1. Data Denoising

To ensure the high quality and accuracy of the ECG signal data extracted from the PTB-XL database, we performed a multi-step data cleaning and denoising process. The goal was to preprocess the ECG signals to eliminate noise, external disturbances, and other artifacts. The following outlines the specific procedures and methods employed: Firstly, to reduce high-frequency noise in the signals, we applied a smoothing filter. This filter smooths the signal by calculating the average value within a fixed window. Next, to address the issue of power line interference, we used a notch filter. Power line interference, introduced by the frequency of the power system, appears as periodic interference in ECG signals. The notch filter effectively suppresses this specific frequency interference. Baseline drift, a common low-frequency interference typically caused by patient respiration or poor electrode contact, was removed using a high-pass filter.

During the preprocessing phase, we conducted comprehensive experiments to select the most effective window functions and cutoff frequencies for these filters, ensuring that the methods were well-suited for ECG signal denoising. These optimized techniques provided high-quality data for subsequent HRV extraction. By employing these methods, we enhanced the ECG signal quality, ensuring that the extracted HRV data would be accurate and reliable for the further analysis and classification of heart diseases.

### 4.2. HRV Signal Extraction

HRV data are obtained by calculating the distance between ECG signal adjacent R peaks, indicating the variations between successive heartbeats. The standard ECG components include P waves, QRS complexes, and ST segments, all of which can represent different cardiac activity. The QRS complex represents electrical changes during ventricular depolarization, while the RR interval, marked by the peaks of two consecutive R waves, is commonly used to detect arrhythmias.

**Figure 2:**
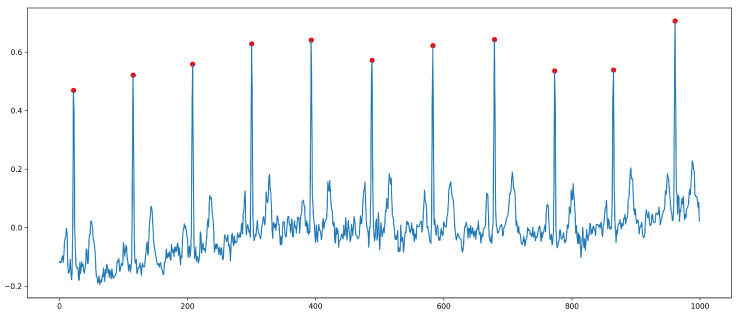
R-peak detection.

Accurate identification of each R peak of the ECG signal is critical for extracting HRV signals, as it provides a reliable foundation for further calculations. Generally, HRV signal extraction involves two primary steps: first, identifying each R peak in the raw ECG signal, and then calculating the intervals between each R peak to generate the HRV signal. Figure 2 illustrates the results of R-peak (red points) identification.

### 4.3. HRV Signal Calculation Method

In the process of detecting the R peaks in ECG signals, the presence of certain measurement errors may affect the integrity of the QRS waveform and thereby impact the accurate identification of the R peak. To address this issue, the R-peak identification method utilized in this study can effectively extract R peaks under various conditions, enhancing the accuracy and stability of R-peak recognition. As shown in Figure 2, in this scenario, we did not detect any instances that did not meet the R-peak criteria. In the subsequent interval calculation process, inappropriate adjacent R-peak time intervals are processed based on the set threshold, ensuring that the extracted HRV signals more accurately reflect the actual situation. The HRV signal calculation parameter notations are shown in Table 2.

HRV is calculated by detecting the R peaks as follows:

(1) $$Y_{c_{i}}=X_{c_{i+1}}-X_{c_{i}}$$

where $Y_{c_{i}}$ is the difference between two adjacent signal points and $X_{c_{i}}$ is the i-th signal point. The calculation formula for max_index is as follows:

(2) max_index=argmax(E[j],I≤j≤I+W)+I

where max_index is the index of the local maximum found within a specified window. E is the array of signal data, I marks the starting index, and W marks the window size. The calculation formula for *T* is as follows:

(3) $$T=0.6\times \left(V_{\mathrm{MAX}}-V_{\mathrm{MIN}}\right)+V_{\mathrm{MIN}}$$

where the gap between $V_{\mathrm{MAX}}$ and $V_{\mathrm{MIN}}$ is calculated and multiplied by 60%. The final value plus the minimum value, $V_{\mathrm{MIN}}$, is *T*. The calculation formula for $C_{i}$ is as follows:

(4) $$C_{i}=\max\left\{E_{I-1},E_{I},E_{I+1}\right\}\;and\;C_{i}>T$$

where $C_{i}$ represents the value at position *I* in the signal data *E* that meets the condition of being greater than the threshold *T*. $\max\{E_{I-1},E_{I},E_{I+1}\}$ finds the local maximum in the range from I−1 to I+1. The calculation formula for $P_{i}$ is as follows:

(5) $$P_{i}-M_{LAST}\leq D_{MAX}$$

where $P_{i}$ represents the position of the *i*-th element in the peaks array, $M_{\mathrm{LAST}}$ is the last element’s position in the merged peaks array, and $D_{\mathrm{MAX}}$ is the maximum distance. The formula to compute *L* is:

(6) $$L=[R_{i+1}-R_{i}|i=0,1,\ldots ,\mathrm{LEN}\left({\mathrm{RPEAK}}_{\mathrm{INDEX}}\right)-2]$$

where *L* is the processed HRV signal, the position of the *i*-th peak is $R_{i}$, and $\mathrm{LEN}({\mathrm{RPEAK}}_{\mathrm{INDEX}})$ is the length of the R-peak position array.

### 4.4. Rule Extraction

In the human nervous system, different frequency bands of HRV correspond to different nervous systems, and these systems have different activity responses to HRV. Therefore, these need further processing after extracting the HRV signal. Based on this, we conducted deeper processing of the HRV signals to more accurately evaluate these physiological indices.

After calculating the spectral data using STFT, the data were divided into three different spectral components: the High-Frequency spectrum 2 (HF2), covering 0.25–0.4 Hz, the High-Frequency spectrum 1 (HF1), covering 0.15–0.25 Hz, and the Low-Frequency (LF) spectrum, covering 0–0.15 Hz. The frequency segment grading is shown in Table 3.

**Figure 3:**
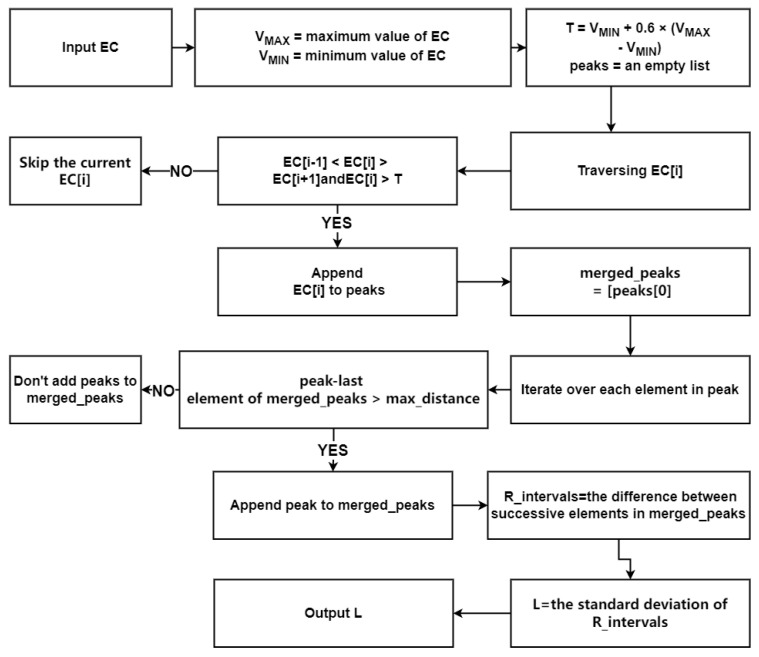
HHDR model—HRV signal extraction algorithm flowchart.

Some specific steps of the calculation process of the HRV signal calculation method are as follows (Algorithm 1); the Algorithm 1 calculation flowchart is shown in Figure 3.
**Algorithm 1** HHDR Model—HRV Signal Extraction Algorithm**Input:** EC**Output:** L1 : **Begin**2 : Initialize parameters3 : $V_{\mathrm{MAX}}\leftarrow$ the maximum value of EC4 : $V_{\mathrm{MIN}}\leftarrow$ the minimum value of EC5 : $T\leftarrow V_{\mathrm{MIN}}+0.6\times \left(V_{\mathrm{MAX}}-V_{\mathrm{MIN}}\right)$6 : peaks ← an empty list7 : **for** *i* **from** 1 **to** len(EC) - 2 **do**8 :   **if** EC[i−1]<EC[i]>EC[i+1] **and** EC[i]>T **then**9 :       Append *i* to peaks10 :    **end if**11 : **end for**12 : merged_peaks ←[peaks[0]]13 : **for each** peak **in** peaks[1:] **do**14 :    **if** peak - last element of merged_peaks > max_distance **then**15 :       Append peak to merged_peaks16 :    **end if**17 : **end for**18 : R_intervals ← the difference between successive elements in merged_peaks19 : L← the standard deviation of R_intervals20 : End

After decomposing the spectral components, we used Spectral Magnitude Quantification (SMQ) to extract features from each frequency band’s spectral component. Since different HRV signal spectral components reflect different heart activities, the features obtained also vary. First, we performed graded quantification of the values, dividing them according to the maximum signal amplitude in each band: “A” level is 0–25%, “B” level is 25–50%, “C” level is 50–75%, and “D” level is 75–100%. According to the above classification rules, we performed spectral magnitude quantification extraction, and the sequence is obtained by matching after extraction.

Based on the previously mentioned methodology, to analyze the most representative spectral magnitude quantization sequences for each type of heart disease, we represent each lead as a sequence segment. We use a sliding window on 12 Sequence Segments (SS) with window lengths of 2, 3, and 4, which were separately generated by sliding window lengths 2, 3, and 4 HRV signal spectral magnitude quantization sequences points. By applying windows of different lengths, we sequentially compute multiple combined sequences of HRV signal bands for each segment, further extracting the combined sequence rules for each type of heart disease.

In calculating the aforementioned combined sequence rules, we used the FP-growth calculation method to mine the frequent sequential rules. Initially, for each patient, the 12-lead HRV signals are divided into sequence segments according to each lead. We calculate the frequency of each sequence for each type of heart disease, exclude combinations with relatively low frequencies, and retain the more frequently occurring sequence combinations. Next, we generate an association rule set from the retained segment combinations and evaluate the reliability of the new combinations, eliminating those with low reliability. The support confidence calculated for each group is used as a basis for reliability, and these are used as a basis for closeness of the rules. This process is repeated for each sequence segment.

**Figure 4:**
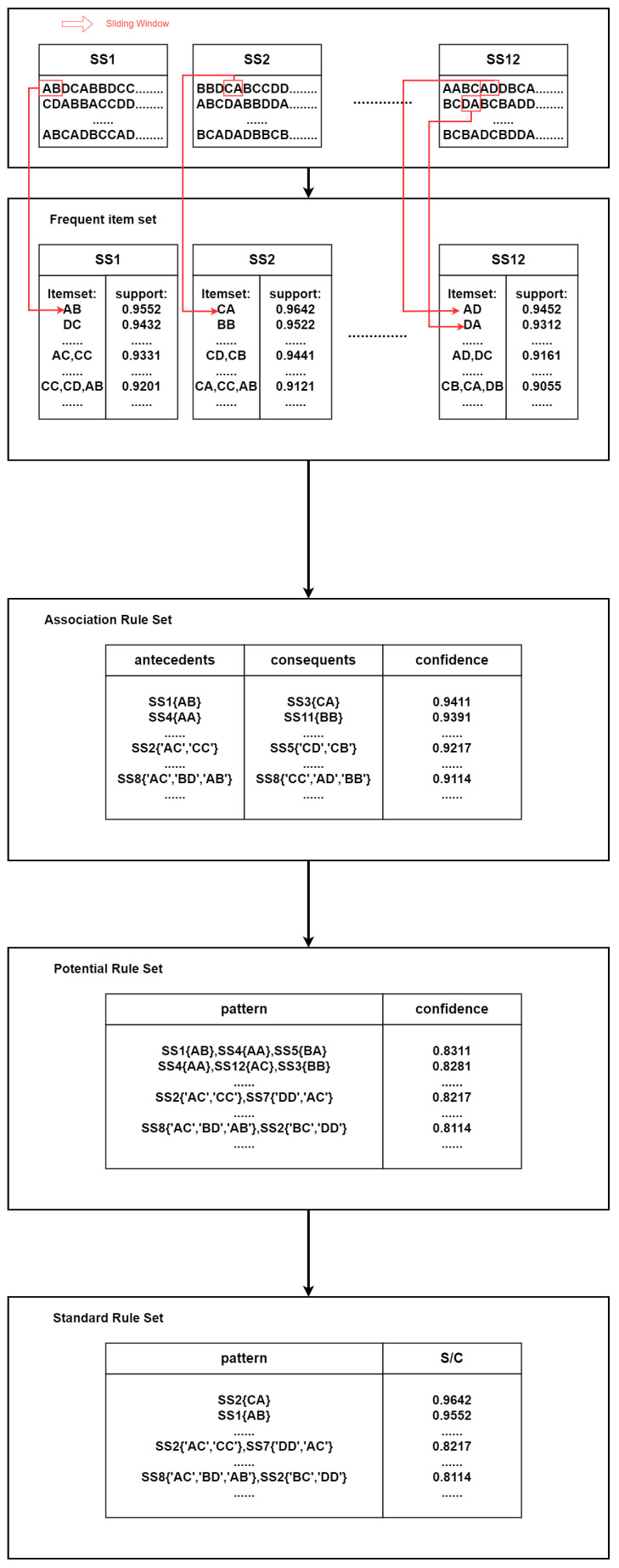
Rule extraction example.

Finally, we calculate the correlation values between adjacent sequence segments in the association rule set, which indicate the simultaneous occurrence of a sequence in both segments and the likelihood of their co-existence. If the correlation between two sequence segments is high and the preceding combination is present in the current rules, it is set as a prefix and suffix relationship. The generated prefix–suffix relationship sequences are classified into a potential rule; its support value is the prefix support multiplied by the correlation value. These potential rule sets, along with the frequent item sets, form a standard rule set. Figure 4 provides an example of this process, demonstrating the sequence rules (such as AB, DC, et al.) with a window length of 2 (red boxes). The figure demonstrates that the standard rule set possesses significant strength, representing its applicability to each heart disease.

**Figure 5:**
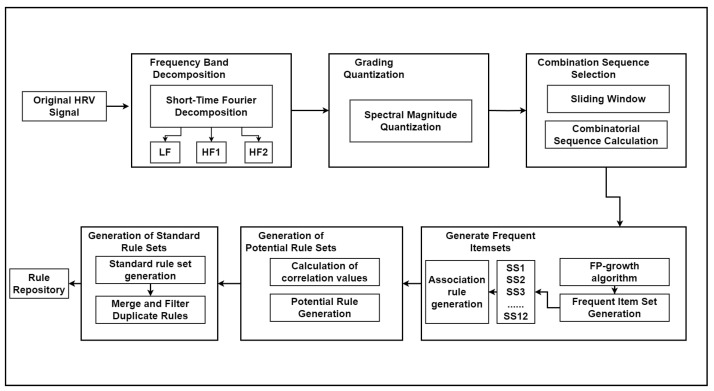
Rule extraction process workflow.

Since the rule sets for each type of heart disease may contain identical rules, the same rules in the rule set may have effects on different classes, and these rules will be discarded in the subsequent matching task. Therefore, in our experiments we eliminate duplicates from the rule sets for each category of heart disease, retaining unique rules and filtering out common rules. Figure 5 presents the workflow diagram for the rule extraction process.

### 4.5. Matching Rule Establishment Algorithm

The parameter expressions of the rule extraction calculation method are shown in Table 4.

Using the Short-Time Fourier Transform (STFT) technique for frequency band decomposition, the formula is presented below:

(7) MHRV=RebuildSTFT(WaveSTFT(WHRV)

where MHRV is the HRV signal after frequency band decomposition, WHRV is the HRV signal sequence, and RebuildSTFT and WaveSTFT are, respectively, the short-time Fourier transform’s frequency band decomposition and the reconstruction functions. The formula for quantifying spectral amplitude values is outlined below:

(8) $$MQA=\left\{\begin{array}{c} A,|QE|\in (0,0.25\times A_{\max})\\ B,|QE|\in (0.25,0.5\times A_{\max})\\ C,|QE|\in (0.5,0.75\times A_{\max})\\ D,|QE|\in (0.75\times A_{\max},A_{\max}) \end{array}\right.$$

where MQA represents the Magnitude Quantization Levels, Qe is the amplitude value, and Amax indicates the peak absolute value in the intensity series. The method to generate frequent item sets is as follows:

(9) FQ=fp(MQA)

where FQ is the frequent item set; the fp function is utilized to extract frequent item sets. The core calculation formula for support is as follows:

(10) $$Support\left(X\right)=\frac{TransactionscontainingX}{TotalTransactions}$$

where TransactionscontainingX indicates the number of records that include itemsetX. TotalTransactions represents the total count of transactions. Support(X) indicates the support of itemsetX. The expression for association rule mining is given below:

(11) AQA=A_Rules(FQ)

where AQA is the set of potential rules; the A_Rules function extracts the association rule set from frequent item sets. The expression for calculating confidence is as follows:

(12) $$CF\left(X\to Z\right)=\frac{SP(X\cup Z)}{SP(X)}$$

where CF(X→Z) represents the confidence of the rule X→Z. SP(X∪Z) indicates how often both item sets X and Z appear together in transactions. SP(X) denotes the support for item set X. The following expression is the expression for mining the latent rule set:

(13) RN=Mine(FQ,AQA)

where RN is the combined rule set; the mining method extract the set of potential rules from frequent item sets and association rule sets, generating a standard rule sets. The following expression is about rule deduplication:

(14) TNA=RemoveDuplicates(RN)

The RemoveDuplicates function is used to remove duplicate rules. The deduplication process may involve comparing the antecedent and consequent parts of rules to ensure each rule is unique.

**Figure 6:**
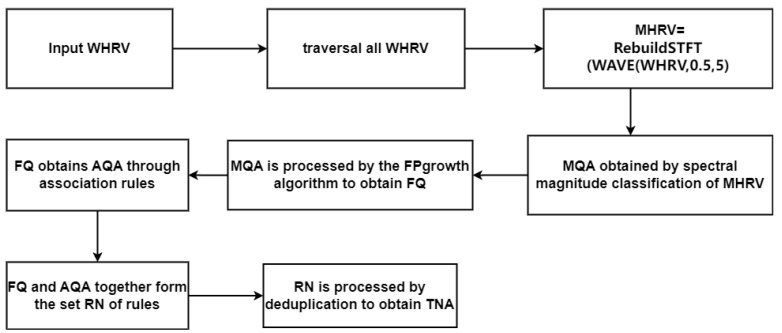
HHDR model—Matching rule mining algorithm flowchart.

The specific content of the algorithm for extracting rules is as shown in the following (Algorithm 2); the Algorithm 2 calculation flowchart is shown in Figure 6.
**Algorithm 2** HHDR Model—Matching Rule Mining Algorithm**Input:** WHRV**Output:** TNA1 : **Begin**2 : **for** all WHRV **do**3 : MHRV = RebuildSTFT(WAVE(WHRV,0.5,5))4 : the sequence MQA is obtained by Spectral Magnitude classification of MHRV5 : **end for**6 : MQA label clustering7 : **for** each of labels **do**8 : FQ = fp (MQA)9 : AQA = A*_Rules_* (FQ)10 : RN = Mine (F1, AQA)11 : TNA = RemoveDuplicates(RN)12 : **end for**

### 4.6. Extraction of Spectral Magnitude Quantization Algorithm Features

**Figure 7:**
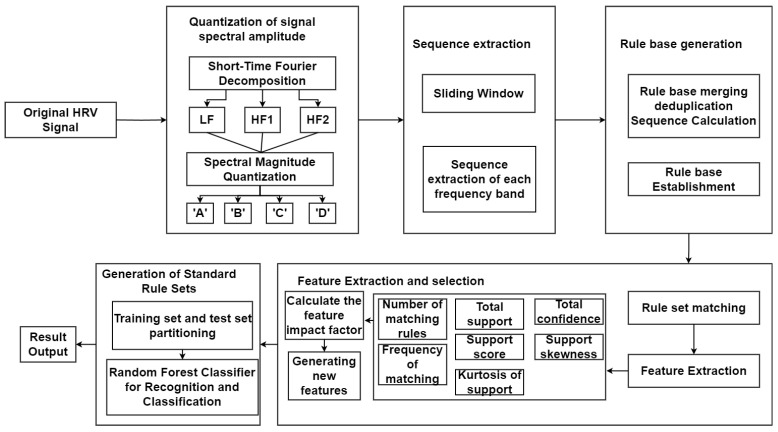
Spectral magnitude quantization feature extraction process.

In the process of using Heart Rate Variability (HRV) for heart disease recognition, feature extraction is a critical step, accounting for a significant proportion of research. This study focuses on extracting the Spectral Magnitude Quantization (SMQ) features based on the extracted rule set of all heart disease rules, ‘A’, ‘B’, ‘C’, ‘D’ are the spectral magnitude quantization levels that caclulated by formula 8, as shown in Figure 7. This approach to feature extraction ensures a reliable basis for the high-accuracy matching tasks that follow.

Due to the independence of HRV signals in each lead and each frequency band of the lead, the obtained spectral magnitude sequences differ. Therefore, a sliding window method is employed to extract spectral magnitude sequences under different windows for each frequency band of each lead. These extracted sequences are subsequently matched to the rule set. Regarding the three HRV frequency bands, seven features are extracted: the number of matching rules, total support, total confidence, matching frequency, support score, support skewness, and support kurtosis.

After feature extraction, impact factor calculation is performed on 21 features across the three frequency bands for various heart diseases. The top three features influencing each type of disease are selected, and five new features are generated by combining these selected features. These five new features, along with the top three influencing features of each disease, are then input into a random forest model for training and recognition. The workflow diagram for this process is illustrated in Figure 7.

### 4.7. Spectral Magnitude Quantization Algorithm

The spectral magnitude quantization algorithm’s parameter notations are shown in Table 5. MR indicates the frequency of each quantized sequence aligning with the rule set, and the formula is given below:

(15) $$MR=\left\{\begin{array}{cc} \mathrm{MR}+1, & {\mathrm{MQA}}_{i}={\mathrm{TNA}}_{i}\\ \mathrm{MR}, & {\mathrm{MQA}}_{i}\neq {\mathrm{TNA}}_{i} \end{array}\right.$$

where MQA is the spectrum amplitude quantization sequence extracted using a sliding window, TNA is a rule in the rule library, and the calculation process of TC is given below:

(16) $$TS=\left\{\begin{array}{cc} {\mathrm{TS}+\mathrm{SQ}}_{i}, & {\mathrm{MQA}}_{i}={\mathrm{TNA}}_{i}\\ \mathrm{TS}, & {\mathrm{MQA}}_{i}\neq {\mathrm{TNA}}_{i} \end{array}\right.$$

where $SO_{i}$ represents the support value of each rule within the set. The calculation formula for overall confidence, TC, is given below:

(17) $$TC=\left\{\begin{array}{cc} {\mathrm{TC}+\mathrm{CQ}}_{i}, & {\mathrm{MQA}}_{i}={\mathrm{TNA}}_{i}\\ \mathrm{TC}, & {\mathrm{MQA}}_{i}\neq {\mathrm{TNA}}_{i} \end{array}\right.$$

where $CO_{i}$ represents the confidence value of the current rule within the set. Calculation formula for frequency, FR, is given below:

(18) $$FR=\frac{MR}{LE}$$

where LE denotes the total sequences obtained through the sliding window for spectral amplitude quantization. The calculation formula for the support score, SQ, is given below:

(19) $$SQ=\left\{\begin{array}{cc} {\mathrm{SA}}_{\left[\frac{i+1}{2}\right]}, & i=a\\ \frac{{SA}_{\left[\frac{i}{2}\right]}+{SA}_{\left[\frac{i+1}{2}\right]}}{2}, & i\neq a \end{array}\right.$$

where $SA_{i}$ is the support value of the *i*-th matching sequence, and a is an odd number. The calculation formula for the support skewness, SS, is given below:

(20) $$SS=\frac{n}{\left(n-1)(n-2\right)}\sum_{i=1}^{n}{\left(\frac{x_{i}-\bar{x}}{s}\right)}^{3}$$

where *n* represents the count of support matches matched by the sequence, *x* is the *i*-th value in the support data matched by the sequence, $\bar{x}$ is the mean of the support data matched by the sequence, and *s* is the standard deviation of the support values matched by the sequence. Calculation formula for support kurtosis, SK, is given below:

(21) $$SK=\frac{n(n+1)}{\left(n-1)(n-2)(n-3\right)}\sum_{i=1}^{n}{\left(\frac{x_{i}-\bar{x}}{s}\right)}^{4}-\frac{3{\left(n-1\right)}^{2}}{\left(n-2)(n-3\right)}$$

where *n* represents the count of support matches matched by the sequence, $x_{i}$ is the *i*-th value in the support data matched by the sequence, $\bar{x}$ is the mean of the support data matched by the sequence, and *s* is the standard deviation of the support values matched by the sequence. Calculation formula for the newly generated feature, $FN_{\mathrm{NCMSH}}$, is given below:

(22) $$FN_{\mathrm{NCMSH}}=\frac{\sum_{i=1}^{n}W_{i}x_{i}}{\sum_{i=1}^{n}W_{i}}$$

where $FN_{\mathrm{NCMSH}}$ represents the collective term for the five new features in the data, *w* denotes the feature weight, and *x* is the *i*-th feature value. After feature extraction finishes, they are processed by a random forest classifier for training and recognition. This formula is given below:

(23) $$AC=\mathrm{RandomForestClassifier}(OF,FN_{N},FN_{C},FN_{M},FN_{S},FN_{H})$$

where RandomForestClassifier represents the classification using the random forest algorithm. AC is the accuracy of the classifier.

**Figure 8:**
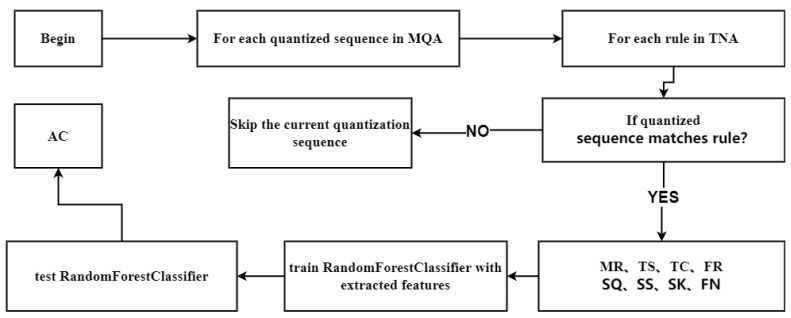
HHDR model—Spectral magnitude quantization algorithm flowchart.

The following are some specific algorithms in the calculation process of HRV signals (Algorithm 3); the Algorithm 3 calculation flowchart is shown in Figure 8.
**Algorithm 3** HHDR Model—Spectral Magnitude Quantization Algorithm**Input:**MQA, TNA**Output:**AC1: **Begin**2: **for** each quantized sequence in MQA **do**3:  **for** each rule in TNA **do**4:   if quantized sequence matches rule then5:    increment MR by 16:   **end if**7: **end for**8: **for** each rule in TNA **do**9:    TC = TC + CO_*i*_10:  TS = TS + SO_*i*_11: **end for**12: FR = MR / LE13: SQ = SQ + SA_*i*_14: SS = SS + ${\left(\frac{x_{i}-\bar{x}}{s}\right)}^{3}$15: SS = SS * $\left(\frac{n}{\left(n-1)\cdot (n-2\right)}\right)$16: **for** i = 1 to n **do**17:  SK = SK + ${\left(\frac{x_{i}-\bar{x}}{s}\right)}^{4}$18: **end for**19: **for** each feature in FNNCMSH **do**20:  FN = FN + w·x21: **end for**22: train RandomForestClassifier with extracted features23: test RandomForestClassifier24: AC = calculate final accuracy

## 5. Experiment and Results

### 5.1. Dataset

We chose the PTB-XL large-scale ECG dataset in our experiments [27]. This dataset includes multiple diagnostic categories, making it a multi-label dataset. The NORM class has the highest number of instances, while the HYP class has the fewest. We divide the training to validation ratio of the dataset into 4:1. Section 3.2 provides a detailed introduction to the dataset.

### 5.2. HHDR Model Evaluation

**Figure 9:**
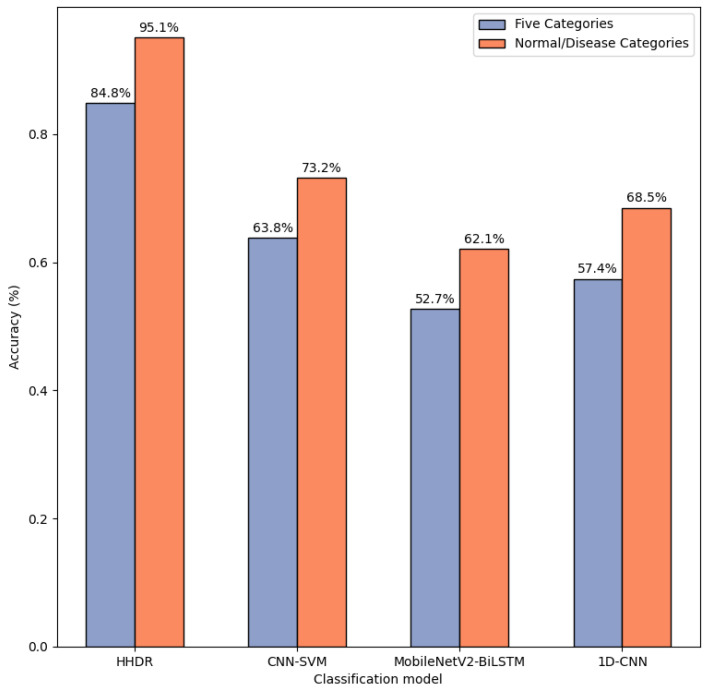
Classification model accuracy results.

After the above research process, we evaluated the HHDR model, and the obtained conclusion indicates that it is valid in recognizing both normal/disease categories and five types of heart diseases. On the PTB-XL dataset, we selected multiple models for testing comparisons, including the CNN-SVM model [28], the MobileNetV2-BiLSTM model [29], and the 1D-CNN model [30]. In these models, the performance of the HHDR model is remarkable. The detailed experimental results are shown in Figure 9.

**Figure 10:**
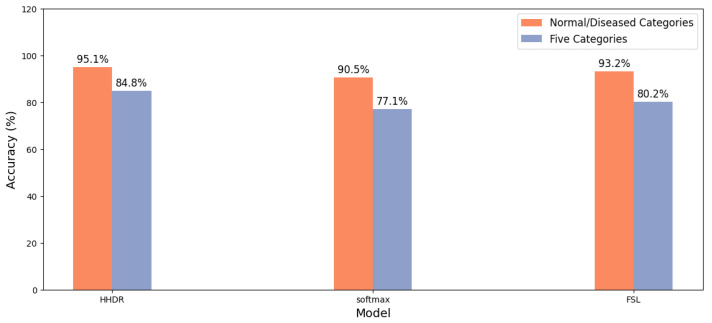
Model comparison diagram.

In this paper, our model (HHDR) compares the performance of two SOTA models (softmax [15], FSL [16]). The models are evaluated on two different classification tasks, namely normal/lesion category and five-class classification. This is illustrated in Figure 10, which shows the percentage performance of each model on both tasks.

In the selection of classification models, we compared classical classification models for normal/disease identification and five-class classification. The models compared are Naive Bayes Classifier (NBC), K-Nearest Neighbors (KNN), Random Forest (RF), and Support Vector Machine (SVM). In normal/disease identification, NBC achieved 70.1% accuracy, SVM 73.3%, KNN 80.8%, and RF 95.1%.

**Figure 11:**
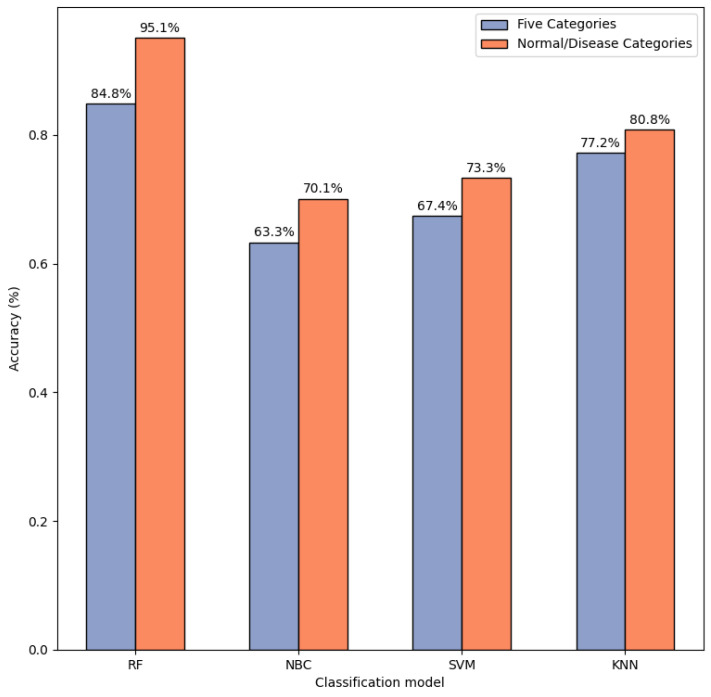
Five-category classification model comparison.

The accuracies for five-class classification were 63.3% for NBC, 67.4% for SVM, 77.2% for KNN, and 84.8% for RF. Regardless of whether it is normal/disease identification or five-class classification, the RF model performs better than the other models. Therefore, we selected the random forest model as the classifier. In Figure 11, the results of the experiment are presented.

Our research model, HHDR, is particularly notable in feature extraction. We extracted features from three frequency bands, with seven features per band. Based on these features, we calculated the impact factor of each feature for each category, then combined the top three features with the highest impact factors for each category. Compared with traditional feature extraction methods, we extracted the temporal features, frequency domain features, and nonlinear features.

We are interested in evaluating the effect of the SMQ feature extraction method on different classification algorithms, so we conducted comparative experiments using four classifiers: NBC, KNN, SVM, and RF. The experimental results indicate that in the classification of normal versus disease states, the highest accuracy of time domain feature recognition is 66.3%, frequency domain feature recognition is 64.9%, and HRV nonlinear feature recognition is 65.2%. The accuracy of the SMQ features extracted in our experiment is 95.1%, significantly higher than other features.

In five-class classification, the highest accuracy of time–frequency feature recognition is 57.6%, frequency domain feature recognition is 50.6%, and HRV nonlinear feature recognition is 53.1%. The accuracy of the SMQ features extracted in our experiment is 84.8%, significantly higher than other features.

**Figure 12:**
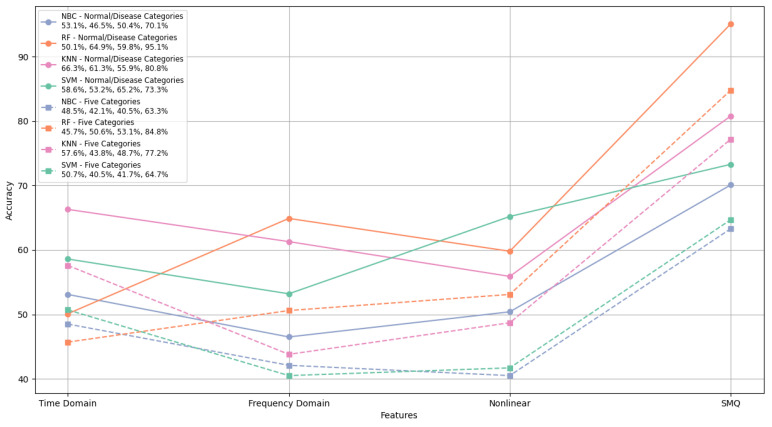
Feature extraction algorithms comparison.

As shown in the figure, the random forest classification model performs the best in both normal/disease classification and five-class classification. At the same time, the random forest classification model also performs well in frequency domain features, indicating that the random forest classification model is better suited for frequency-related feature classification and recognition. The experiments demonstrate that the SMQ feature extraction algorithm outperforms traditional feature extraction models. The results for different features across each model are compared, as shown in Figure 12.

## 6. Discussion

This study demonstrates that utilizing HRV signals for heart disease recognition is feasible. The extracted features, along with those generated based on influence factors, achieved high accuracy in both normal/disease recognition and five-category classification. Our proposed SMQ feature extraction algorithm is not similar to the usual feature extraction methods, because, except for the extracted 21 basic features for three bands, and 5 kinds of diseases’ features based on specific rule sets, our constructed rule sets are the rule-based features’ sequences, which mean the rule-based features sequences set. Moreover, most of the existing methods’ extracted features are status features for certain conditions, not considered the “changes” status. Our method also calculates the “changes” features, which makes it easier to track the continuously generated heart status in order to improve the heart diseases recognition accuracy rate.

Preliminary data cleaning, R-peak extraction, and subsequent feature selection provided a solid foundation for high accuracy. To ensure the high quality and accuracy of the extracted ECG signal data, we performed a multi-step data cleaning and denoising process, as described in Section 4.1. The aim was to preprocess the ECG signal before extracting the R peak to eliminate noise, external interference, and other artifacts. In our feature extraction process, the SMQ feature extraction method was crucial. By designing and mining association rules and generating rule sets, we removed redundant rule sets, ensuring the uniqueness of rule sets for each category, which was essential for subsequent matching work. Additionally, we performed mining in three bands, where each band represented different indicators, which is a critical step. The random forest algorithm demonstrated superior performance, especially in normal/disease recognition, compared to other algorithms.

The classification of heart disease based on HRV signals is significant for health monitoring. By extracting and analyzing these signals, heart diseases can be effectively monitored and identified. During the experiment, we found that the HYP category had lower performance metrics, including accuracy, compared to the other four categories, while the NORM category had the highest performance metrics. On the PTB-XL dataset, reviewing the references, we discovered that the data volume for HYP was the smallest, significantly lower than that of the other categories, with the largest data volume belonging to the NORM category.

The HRV signal features identified in this study allow for high-accuracy recognition and classification. The primary advantage is that HRV data can be obtained by analyzing Electrocardiogram (ECG) signals, covering multi-dimensional features from the time domain to the frequency domain, reflecting various aspects of heart activity. Accordingly, the advantage of the proposed SMQ algorithm lies in its ability to mine specific fluctuation patterns between different heart diseases and decompose these patterns into different frequency ranges. Furthermore, different frequency ranges of fluctuations represent different physiological responses, illustrating the physiological activity forms of the heart. These differential frequency band features can be used for heart disease classification, with experimental results validating the effectiveness of this method. Despite the successful HRV spectral analysis using the PTB-XL dataset in this study, we must admit that the length of each record in the dataset is 10 s, which brings certain limitations to the traditional HRV spectral analysis. Standard HRV analysis usually requires a long time window. To cope with this limitation, we adopt a multi-lead data approach, where the data of 12 leads are divided into 12 matched segments and analyzed as a whole rather than as a single lead. At the same time, making full use of the characteristics of short-time data that can sharply capture the changes of high-frequency components, each short-time data window is divided into a low-frequency and two high-frequency intervals for analysis. Future research will investigate the link between HRV signals and cardiac diseases to enhance the accuracy and consistency of cardiac disease classification. Additionally, we will examine how combining HRV signals with other physiological indicators affects cardiac disease identification, aiming to enhance accuracy and reliability. This approach has broad application prospects. Employing HRV signals for cardiac disease identification can facilitate the timely diagnosis of a patient’s cardiac health and help patients understand the heart status in time.

## 7. Conclusions

This study is a further investigation into electrocardiogram signals, using Heart Rate Variability (HRV) signals computed from ECGs to further analyze and classify cardiac diseases. The experimental results in the field of cardiac disease identification based on HRV signals showed an accuracy of 95.1% for normal/disease classification and 84.8% for classifying five types of cardiac diseases. The experiments confirmed the sensitivity of frequency domain features, including frequency band decomposition, proving them to be effective. Therefore, the HHDR model has demonstrated strong performance in analyzing features related to frequency band decomposition.

## Figures and Tables

**Table 1:** Data distribution table.

Records (Vol.)	Superclass	Description
5469	MI	Myocardial Infarction
9514	NORM	Normal ECG
2649	HYP	Hypertrophy
5235	STTC	ST/T Change
4898	CD	Conduction Disturbance

**Table 2:** HRV signal calculation method notations.

Parameter	Description
Y_*ci*_	Difference from the *i*-th to the subsequent signal points
X_*ci*_	*i*-th signal point
E	Overall signal data array
I	Starting index
W	Window size
T	Threshold
C_*i*_	Value at the i-th position in the signal data E
E_*I*_	*I*-th signal data point
L	HRV sequence signal
R_*i*_	*i*-th peak position
P_*i*_	*i*-th element in the peak value list position
D_*MAX*_	Maximum distance
V_*MAX*_/V_*MIN*_	Maximum value/Minimum value
M_*LAST*_	Position of the last element in the merged peak value list
max{$E_{I-1}$, $E_{I}$, $E_{I+1}$}	Maximum value in E within the range from I − 1 to I + 1
LEN(${\mathrm{RPEAK}}_{\mathrm{INDEX}}$)	Length of the R-peak position list
max_index	Index position of the local maximum
EC	Original ECG signal

**Table 3:** Frequency segment grading.

Frequency Segment	Frequency Span
HF2	0.25–0.4 Hz
HF1	0.15–0.25 Hz
LF	0–0.15 Hz

**Table 4:** Frequency band grading parameters.

Parameter	Description
MHRV	Frequency-divided HRV signal
WHRV	HRV signal sequence
MQA	Magnitude Quantization Levels
RebuildSTFT	STFT-based reconstruction function
WaveSTFT	STFT-based decomposition function
Qe	amplitude value
Amax	absolute value of the peak in the amplitude series
FQ	frequent itemset
AQA	potential rule set
RN	merged rule set
TNA	deduplicated rule set

**Table 5:** HRV signal extraction algorithm notations.

Parameter	Description
MR	Number of Matched Rules
MQA*_i_*	The *i*-th spectral magnitude quantization sequence in the sequence
TNA*_i_*	*i*-th rule in the set
TS	Overall support
SO*_i_*	Rule i support
TC	Overall confidence
CO*_i_*	Rule i confidence
FR	Frequency
LE	Sliding window extraction of spectral magnitude quantifying sequences
SQ	Support score
FN_NCMSH_	The collective term for the five newly generated features
SS	Support skewness
s	The standard deviation of the support of matching sequences
SK	Support kurtosis
AC	Accuracy in feature recognition classification
OF	Original features
${\mathrm{SA}}_{\left[i\right]}$	The support of the *i*-th matching sequence

## Data Availability

A publicly available dataset was analyzed in this study. This data can be found here: https://www.physionet.org/content/ptb-xl/1.0.3/(accessed on 9 November 2022).
